# General principles of binding between cell surface receptors and multi-specific ligands: A computational study

**DOI:** 10.1371/journal.pcbi.1005805

**Published:** 2017-10-10

**Authors:** Jiawen Chen, Steven C. Almo, Yinghao Wu

**Affiliations:** 1 Department of Systems and Computational Biology, Albert Einstein College of Medicine, Bronx, New York, United States of America; 2 Department of Biochemistry, Albert Einstein College of Medicine, Bronx, New York, United States of America; 3 Department of Physiology and Biophysics, Albert Einstein College of Medicine, Bronx, New York, United States of America; Rutgers University, UNITED STATES

## Abstract

The interactions between membrane receptors and extracellular ligands control cell-cell and cell-substrate adhesion, and environmental responsiveness by representing the initial steps of cell signaling pathways. These interactions can be spatial-temporally regulated when different extracellular ligands are tethered. The detailed mechanisms of this spatial-temporal regulation, including the competition between distinct ligands with overlapping binding sites and the conformational flexibility in multi-specific ligand assemblies have not been quantitatively evaluated. We present a new coarse-grained model to realistically simulate the binding process between multi-specific ligands and membrane receptors on cell surfaces. The model simplifies each receptor and each binding site in a multi-specific ligand as a rigid body. Different numbers or types of ligands are spatially organized together in the simulation. These designs were used to test the relation between the overall binding of a multi-specific ligand and the affinity of its cognate binding site. When a variety of ligands are exposed to cells expressing different densities of surface receptors, we demonstrated that ligands with reduced affinities have higher specificity to distinguish cells based on the relative concentrations of their receptors. Finally, modification of intramolecular flexibility was shown to play a role in optimizing the binding between receptors and ligands. In summary, our studies bring new insights to the general principles of ligand-receptor interactions. Future applications of our method will pave the way for new strategies to generate next-generation biologics.

## Author summary

In order to adapt to surrounding environments, multiple signaling pathways have been evolved in cells. The first step of these pathways is to detect external stimuli, which is conducted by the dynamic interactions between cell surface receptors and extracellular ligands. As a result, recognition of extracellular ligands by cell surface receptors is an indispensable component of many physiological or pathological activities. In both natural selection and drug design, the presence of multiple binding sites in extracellular ligand complexes (so-called multi-specific ligands) is a common strategy to target different receptors on surface of the same cell. Such spatial organization of ligand binding sites can elaborately modulate the downstream signaling pathways. However, our understanding to the interactions between multi-specific ligands and membrane receptors is largely limited by the fact that these interactions are difficult to quantify and they have only been successfully measured in a very small number of cases *in vivo*. Using a simple computational model, we can realistically simulate the binding process between specially designed multi-specific ligands and membrane receptors on cell surfaces. This study therefore provides a useful pathway to unravel basic mechanisms of ligand-receptor interactions and design principles for new drug candidates.

## Introduction

Integral membrane proteins are the sensors of extracellular signals, including cell-cell and cell-substrate interactions, as well as environmental queues. Their interactions with extracellular ligands initiate most of the intracellular signaling pathways [1, 2], while the dysregulation of these receptor-initiated signaling pathways leads to various diseases, such as cancers [3], and greater than 60% of current drugs are designed to target specific cell surface receptors [4, 5]. In many cases, the extracellular ligands are spatially organized into multivalent/multicomponent assemblies. These assemblies, called multi-specific ligands, contain multiple receptor binding sites and are able to target different cell surface receptors simultaneously. For instance, multiple low affinity interactions involving influenza virus hemagglutinin trimers are required for effective recognition of cell surface glycoproteins on bronchial epithelial cells [6, 7]. Another example is the presence of multiple receptor-binding sites in all classes of antibodies (e.g., bivalent, tetravalent and decavalent in IgG, IgA and IgM isotypes, respectively). The overall apparent binding affinity is enhanced due to the synergy between the multiple binding interactions within an immune complex [8], which is commonly referred to as 'avidity' [9]. Although the general properties and biochemical consequences of binding avidity are well appreciated [10], the detailed mechanisms and underlying energetic contributions remain unclear. The importance of several regulatory factors such as the competition between different binding sites and the conformational flexibility in a complex has not been quantitatively evaluated. Moreover, one of the most promising strategies in drug design is the development of synthetic chimeric ligands [11], in which multiple natural ligands are artificially fused to target their cognate receptors on the surfaces of specific cell types. These multi-specific targeting reagents can improve the efficiency and selectivity of drug-based therapies; therefore, enhanced understanding of the basic principles underlying the interactions between multi-specific ligands and their receptors is critical for continued development of new therapeutic strategies.

Computational approaches allow for a wide range of variables to be systematically examined and a variety of different methods have been recently developed to study the interactions between ligands and cell surface receptors. The chemical kinetics of receptor-ligand binding was first described by simple mathematical models [12, 13], and have been improved by consideration of the spatial confinement of membrane receptors [14, 15]. Reaction rates between receptors and ligands were modulated to explore the impact on binding of the reduction in the dimensionality of receptors confined to a two-dimensional bilayer. However, information such as spatial heterogeneity and molecular details were not be captured. In contrast, atom-based molecular dynamic simulations [16] were used to provide full structural descriptions of both ligands and receptors [17–20]. The primary limitation of these atomistic simulations is the large computational overhead, which prohibits these approaches from being applied to multivalent molecular complexes and slower biological processes (i.e., microsecond time scales or longer) [21]. Other hybrid models have been introduced to bridge the gap between mathematical modeling and atomic simulations [22–25], which depend on coarse-grained representations of molecules [26–30], or reduced degrees of freedom in their movements, as captured by lattice-based simplifications [31–33]. For instance, Miguez and colleagues applied Langevin dynamics for ligand-receptor interaction [34], in which ligands and receptors were represented as simplified spherical particles. However, the theoretically “scaled units” used for the simulation parameters in this study are difficult to directly correlated with biological binding properties in a quantitative manner. Moreover, for all the methods described above, the principles of binding avidity between cell surface receptors and multivalent ligands has not been systematically evaluated.

Here we present a computational model to investigate the general mechanism of interactions between membrane receptors and their ligands. The spatial organization of multimeric or multi-domain receptors/ligands is explicitly incorporated into the model. Molecules possessing multiple binding sites are referred as multi-specific receptors/ligands in the following text. In particular, each binding site in a multi-specific receptor/ligand is represented in our coarse grain model as a rigid body, with the binding site explicitly defined on its surface. The overall modeling system contains a large number of individual receptors and ligands, and their diffusion and binding kinetics are simulated by a kinetic Monte-Carlo algorithm. All parameters in the simulation, such as diffusion constants and binding rates, are constrained within biologically relevant ranges. By varying the number of binding sites and the affinity of each binding site, our simulations demonstrate that the overall binding is cooperatively strengthened when multiple binding sites are spatially tethered. Interestingly, this positive coupling effect is reduced in the regime of strong individual binding affinities. Furthermore, by varying the concentrations of receptors on cell surfaces, we illustrate that the cell specificity of ligand binding is highly sensitive to the binding affinity. Finally, by altering the conformational fluctuations within a multi-specific receptor/ligand, we show that molecular flexibility plays an important role in modulating the binding between receptors and ligands. Taken together, our computational model provides insights into both basic mechanisms of ligand-receptor interactions and design principles for new drug candidates. These considerations are especially relevant given the extensive commercial interest in development multi-specific biologics for the treatment of a wide range of clinical indications.

## Model and method

**Fig 1:**
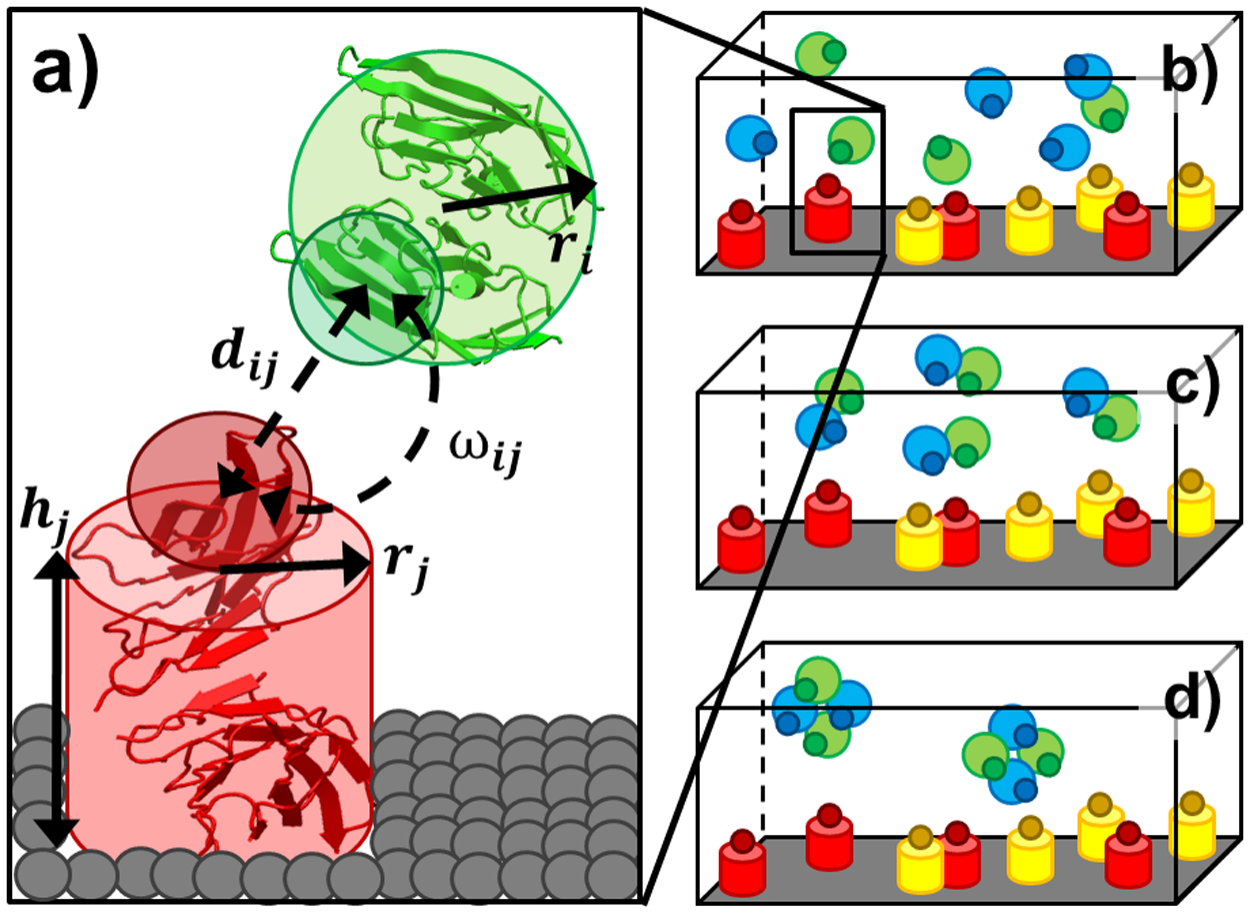
A rigid-body (RB) based model is used to study the interaction between ligands and receptors **(a)**. Each domain or subunit of a ligand is simplified as a spherical rigid body with radius *r*_*i*_. Each receptor is simplified as a cylinder with radius *r*_*j*_ and height *h*_*j*_. A functional site is placed on the surface of each rigid body. The distance between functional sites *d*_*ij*_ and their relative orientation *ω*_*ij*_ need to be below cutoff values to trigger binding reaction between these two molecules. Three scenarios were designed to test the relation between the binding avidity of a multi-specific ligand and the affinity of its individual binding site. In the first scenario, receptors A (red) and C (yellow) are placed on cell surface. Ligands B (green) and D (blue) are separately placed in the 3D extracellular region as monomers **(b)**. In the second scenario, ligand B and D are spatially tethered (referred as *BD*) in the extracellular region **(c)**. In the third scenario, higher-order assembly of a multi-specific ligand is formed, which contains two ligands B and two ligands D (referred as *B*_*2*_*D*_*2*_) **(d)**.

We recently developed a rigid-body (RB) based model to simulate molecular binding in cellular environments [35]. This model has now been enhanced to study the binding interactions between cell surface receptors and soluble ligands. Specifically, the plasma membrane is represented by the bottom surface of a three-dimensional simulation box, the receptor is represented by a rigid body (i.e., cylinder) on the plasma membrane (Fig 1a) and the space above the plasma membrane represents the extracellular region. In the three-dimensional extracellular region, each ligand monomer is simplified as a spherical rigid body with a given radius. To delineate the binding interface, a functional site is defined on the surface of each ligand, as well as the top of each receptor (Fig 1a). Binding between two molecules is triggered by two criteria: 1) the distance between functional sites of two molecules is below a predefined distance cutoff; and 2) the relative orientations of the two engaging molecules fall within specific ranges. In contrast to ligands that can randomly diffuse in bulk solvent with three translational and three rotational degrees of freedom, the diffusion of receptors on the plasma membrane surface are confined. This confinement allows each receptor to rotate only about the axis normal to the plasma membrane, and restricts diffusion to two-dimensional translational movements in the plane of plasma membrane.

To test the relation between the binding avidity of a multi-specific ligand and the affinity of its individual binding sites to receptors, three scenarios were implemented using the above rigid-body model. In the first scenario, receptors A (red) and receptors C (yellow) are placed on cell surface, while ligands B (green) and ligands D (blue) are separately placed in the 3D extracellular region as monomers (Fig 1b). In the second scenario, a ligand B is tethered together with a ligand D in the extracellular region. It is referred as a multi-specific ligand *BD* in the following text. The multi-specific ligand *BD* is represented by two tethered rigid bodies with a binding site of B and a binding site of D on each of their surfaces (Fig 1c). Finally, in the third scenario, a higher-order assembly is represented, which contains two ligands B and two ligands D. This assembly is referred to as the multi-specific ligand *B*_*2*_*D*_*2*_ in the following text. The multi-specific ligand *B*_*2*_*D*_*2*_ is represented by four tethered rigid bodies with two binding sites for B and two binding sites for D on each of their surfaces (Fig 1d). Each multi-specific ligand in the second and third scenarios is simulated as a soluble entity in the extracellular region. Additionally, to capture the contributions of conformational flexibility, binding sites in a multi-specific ligand are allowed to undergo small translational and rotational fluctuations around their mean positions and orientations.

Given the concentration of each molecular species and the type of simulation scenario, the dynamics of the modeling system is simulated by a kinetic Monte-Carlo algorithm, starting from an initial random configuration. In each simulation time step, molecules are first selected at random to model stochastic diffusion; diffusion of membrane-bound receptors are confined to the plasma membrane, while extracellular ligands are free to diffusion throughout the volume of the simulation box. The acceptance ratio of diffusion movements for each molecule is determined by its diffusion coefficient, which is different for soluble ligands and membrane confined receptors. A 2D periodic boundary condition is applied for membrane receptors. In the extracellular region, periodic boundary conditions are imposed along X and Y directions, while in the Z direction, free ligands are not allowed to move below the plasma membrane at the bottom of the simulation volume. Any ligand moving beyond the top of the simulation box is reflected back. Binding is triggered if both distance and orientation criteria between any receptor and ligand are satisfied. The probability to trigger the association is determined by the association rate *k*_*on*_. In contrast, the dissociation between a ligand-receptor pair is described by a probability that is calculated by association rate and binding affinity: $P_{off}=k_{off}\Delta t=C_{0}k_{on}e^{-\Delta G_{0}}\Delta t$, in which *k*_*off*_ is the dissociation rate, Δt is the simulation time step, *C*_*0*_ is the standard unit of concentration and ΔG_0_ is the binding affinity. After a ligand binds to a receptor, the ligand-receptor pair moves as a single unit on plasma membrane. If the ligand contains multiple binding sites, the entire assembly binds together and diffuses with the receptor, such that the remainders of the vacant binding sites in the assembly are accessible for binding by other plasma membrane restricted receptors. The above diffusion-reaction process is iterated until the system reaches equilibrium in both Cartesian and compositional spaces.

The basic simulation parameters, including time step and binding criteria, were adopted from our previous study [35]. Other crucial parameters were chosen from ranges typical for proteins. Each subunit or domain in a multi-specific ligand is represented by a spherical rigid body with radius of 5 nm. For a receptor, the radius of the cylinder is also 5nm, while the height is 10nm. The translation diffusion constant of a soluble ligand monomer is taken as 100μm^2^/s and the rotational coefficient as 5° per ns [36], while the translation diffusion constant of a multi-specific ligand is 50μm^2^/s and the rotational coefficient is 1° per ns. The diffusion of membrane receptors restricted to the plasma surface is much slower, with a translational constant of 10 μm^2^/s and rotational coefficient of 1° per ns. The on-rate for protein association was calibrated to 10^8^M^-1^s^-1^, a relatively high value, to accelerate the simulation. This value is in the typical range of diffusion-limited rate constants, in which association is guided by complementary electrostatic surfaces at binding interfaces [37]. Finally, a wide range of binding affinities, from 5RT to 13RT, was tested, corresponding to dissociation constants between millimolar (mM) and micromolar (μM). Binding of many membrane proteins such as the T-cell receptor (TCR) and T cell co-modulatory molecules are within this range [38]. It is worth mentioning that, although our simulations did not correspond to any specific biological systems due to the lack of sufficient experimental data, it is possible that we can detect some parameters and integrate them into our simulations in the future. For instance, the diffusion constants of membrane receptors on cell surfaces control the kinetics of ligand binding. They can be measured by Total Internal Reflection Fluorescence (TIRF) microscopy by tracking the trajectory of each receptor [39]. Moreover, binding is also affected by the concentrations of ligands and receptors which can be approximately determined by experiments such as flow cytometry [40].

## Results

### Evaluate the relationship between affinity of individual binding sites and overall binding avidity in a multivalent complex

We first investigated how the spatial organization of a multi-specific ligand affects binding between its individual binding sites and their receptors when their affinities are in different ranges. For the spatial organization of a multi-specific ligand, three different simulation scenarios, described in the methods, are used. In all cases, the binding affinities between receptors and ligand binding sites were varied. In order to exclude other factors that can influence binding, such as receptor concentrations in the plasma membrane (i.e., cell surface density), the same size of simulation box and the same number of ligand binding sites were assigned in all three scenarios. Consequently, 100 receptors A and 100 receptors C were placed on a 100nm×100nm cell surface (plasma membrane). In the first scenario, 100 monomer ligands B and 100 monomer ligands D were placed in a 100nm×100nm×50nm cubic box above the cell surface. In the second scenario, 100 tethered ligands *BD* were placed in the box. In the third scenario, 50 assemblies of *B*_*2*_*D*_*2*_ were placed in the box. Therefore, the total number of binding sites, and B and D ligand modules are the same in all there scenarios.

**Fig 2:**
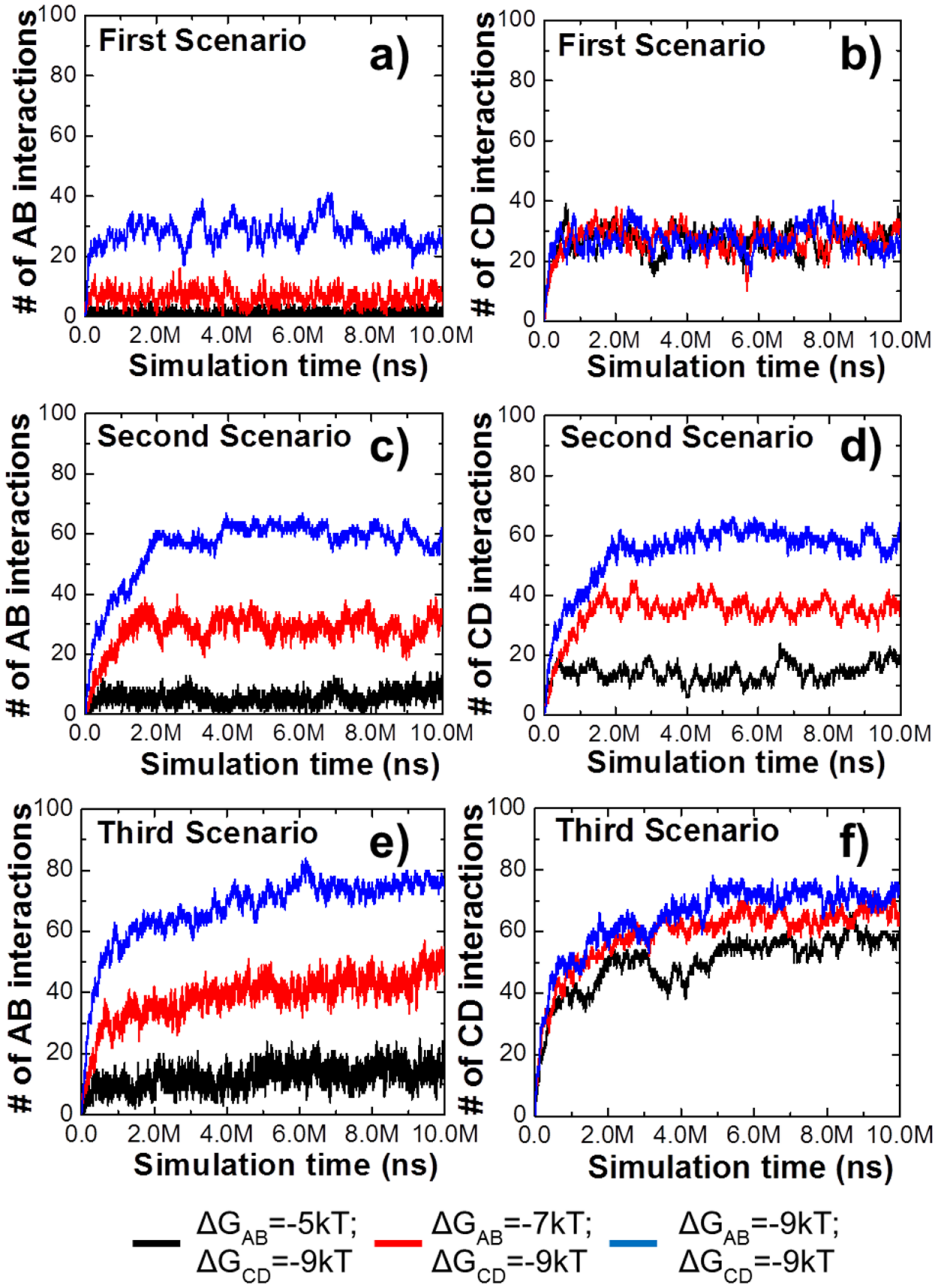
To evaluate how spatial organization of a multi-specific ligand affects its binding with receptors, we fixed the binding affinity between receptor C and ligand D as -9kT. The affinity between receptor A and ligand B were changed from -5kT (black), -7kT (red) to -9kT (blue). The simulation results for the first scenario are shown in **(a)** and **(b)**; the simulation results for the second scenario are shown in **(c)** and **(d)**; and the simulation results for the third scenario are shown in **(e)** and **(f)**. The figure indicates that when ligands B and D are tethered, the interaction between receptor C and ligand D can be affected by the interaction between receptor A and ligand B, although the CD affinity remains unchanged.

The binding affinity between receptor C and ligand D was fixed at -9kT in all three scenarios, while different affinities between receptor A and ligand B were examined. Fig 2a and 2b show the simulation results of the first scenario. In Fig 2a, more interactions between receptor A and ligand B were observed when their binding affinities were stronger. In contrast, the number of interactions between receptor C and ligand D were very close in all simulations, consistent with the invariant binding constant. The results of the second and third scenarios are plotted in Fig 2c to 2f. Similar to Fig 2a, 2c and 2e show more interactions between A and B are formed as the binding affinity increases. However, the numbers of interactions in the second and third scenarios are much higher than the monomer scenario due to the increase of binding avidity. Furthermore, distinct from Fig 2b, 2d and 2f shows that although the affinities between C and D in all simulations are the same, they form very different numbers of interactions. These results indicate that the interaction between receptor C and ligand D can be affected by the interaction between receptor A and ligand B, when ligands B and D are tethered. Overall, our simulations indicated that avidity can enhance binding/occupancy and cause coupling effects between different binding sites.

**Fig 3:**
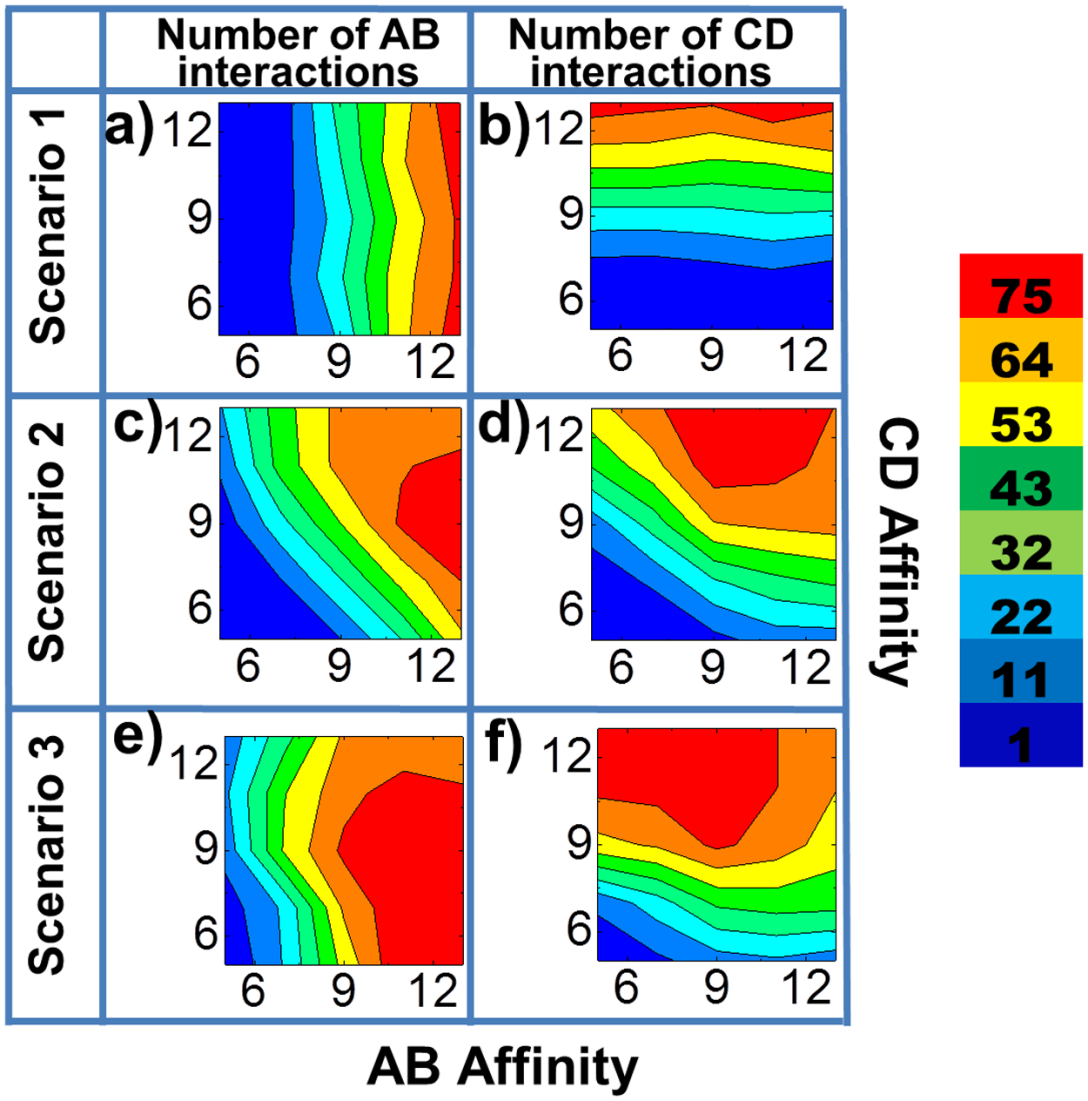
We systematically changed both AB binding affinity and CD binding affinity simultaneously. The overall testing results are plotted as two-dimensional contour profiles. The AB binding affinity is indexed along x axis, while the CD binding affinity is indexed along y axis. The color index of the contours indicates the number of interactions, as shown on the right side of the figure. The numbers of AB interactions formed in the first scenario are illustrated in **(a)** under all combinations of AB and CD affinities, while the numbers of CD interactions are given in **(b)**. For the second scenario, the numbers of AB and CD interactions are recorded in **(c)** and **(d)**, respectively. Finally, the numbers of AB interactions formed in the third scenario are plotted in **(e)** and the numbers of CD interactions are plotted in **(f)**.

To systematically test the effect of avidity and coupling between different binding sites, we simultaneously changed both binding affinities between receptors A and ligands B (AB), and between receptors C and ligands D (CD). The overall results are illustrated in Fig 3a to 3f as two-dimensional contour plots for all three scenarios. The AB and CD binding affinities are indexed along x axis and y axis, respectively. As shown in Fig 3a, when B and D are unlinked, the numbers of AB interactions do not change with CD binding affinity. Similarly, in Fig 3b, the numbers of CD interaction do not change with AB affinity. Therefore, as expected, the binding of ligands B and D with their respective receptors are independent in the first scenario. In contrast, the diagonal distributions of contours in the second scenario (Fig 3c and 3d) suggest that the AB interaction and the CD interaction are correlated with each other. Secondly, comparing with Fig 3a and 3b, the overall contours are shifted to red with the only exception in the high affinity regions. These results demonstrate that if two types of binding sites are tethered in a multi-specific ligand, the binding to their corresponding receptors will be mutually affected (i.e., coupled). The overall binding will be positively enhanced when their individual affinities are not too strong. Moreover, comparing Fig 3e and 3f with Fig 3c and 3d, when ligands B and D are spatially organized as *B*_*2*_*D*_*2*_ in the third scenario, the regions containing largest number of receptor-ligand interactions (the red regions) in their simulated contours are further enlarged. Therefore, the binding between receptors and multi-specific ligands is further strengthened when the avidity of the ligands is increased from *BD* to *B*_*2*_*D*_*2*_. It is notable that if both AB and CD affinities are strong (the upper right corners in Fig 3), the binding of a multi-specific ligand *BD* OR *B*_*2*_*D*_*2*_ with its receptors A and C will be weakened relative to its binding as a monomeric B or D. Possible mechanisms underlying this behavior are considered in the discussions. Finally, the overall binding, the number of both AB and CD interactions, are plotted in S1 Fig as two-dimensional contour plots for all three scenarios under all combinations of AB and CD affinities. The figure shows that there are optimal combinations of AB and CD affinities in the second and third scenarios. The optimal combinations of affinity maximize the total interactions, while these combinations only exist for multi-specific ligands in which binding sites are spatially coupled.

### Quantify the relation between binding affinities of ligands and their binding specificity to cells expressing different numbers of surface receptors

**Fig 4:**
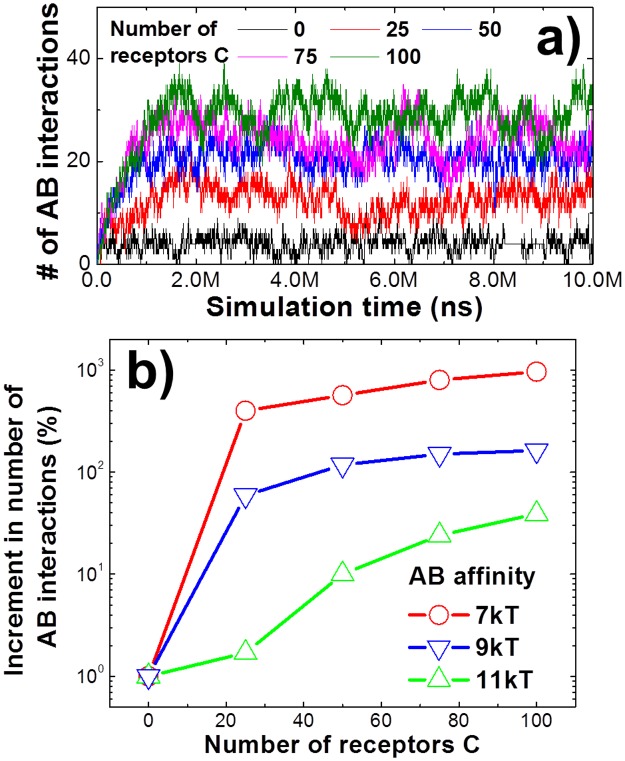
We changed the relative concentrations of two receptors on cell surfaces. The second scenario was applied, in which the total number of receptor A was fixed and the total number of receptor C was changed from 0 to 100. We first fixed both AB and CD binding affinities **(a)**. The figure shows that higher surface densities of receptors C lead to more interactions between receptor A and its ligand. In the second test **(b)**, we changed the affinity between receptor A and ligand B from -7kT to -11kT. The x index of the figure is the number of receptors C on cell surfaces. The relative increment of AB interactions between 0 and a given concentration of receptors C is recorded in the y axis. The simulation results of the figure demonstrate that the ligands with reduced affinity have higher specificity to distinguish different types of cells based on the concentrations of their receptors.

The concentrations of receptors and ligands were fixed in the last section, with the surface density of receptor A equal to that of receptor C. In practice, however, the cell surface expression levels of various proteins vary dramatically. Similarly, the expression levels of a given protein can vary considerably in different cell types. These variations have great functional significance. For example, mutations leading to the overexpression of epidermal growth factor receptor (EGFR) are present in a number of cancer cells and are thought to contribute to the malignant phenotype [41]. Therefore, in this section we examined the consequence of altering the relative concentrations of the two receptors on the plasma membrane. We hypothesize that different concentrations of one receptor type may affect the binding of the other receptor type when their ligands spatially coexist in a single tethered assembly. Specifically, we examined a situation in which the total number of receptor A (100) was fixed in simulations, while the total number of receptor C was varied from 0 to 100. Ligand B and D were tethered as describing in the second scenario. The affinities of both AB and CD interactions were fixed at -7kT and -9kT, respectively. The simulation results, presented in Fig 4a, show that higher surface densities of receptors C lead to more interactions between receptor A and its ligand, although the binding rate and affinity were the same as those used in the original simulations. When no receptor C is present, the number of AB interactions is equivalent to those formed in the first scenario, in which ligand B and D are separated as monomers. In contrast, the presence of more receptors C increases the AB interactions. We speculate that higher surface density of receptors C provides more surface-bound ligand-receptor complexes due to the interaction between receptors C and ligands D. Because ligand B and D are tethered together, the vacant binding sites of ligands B in these surface-bound complexes provide higher local concentration and better orientation to receptors A. These results demonstrate that expression levels of membrane receptors play an important role in regulating the interactions with their multivalent ligands.

In addition to the above simulations, we further changed the affinity between receptor A and ligand B, while maintaining the affinity between receptor C and ligand D. The same simulations were carried out in which ligand B and D are tethered together under different surface densities of receptors C. In short, the concentration of receptor A was fixed, but its affinity with ligand B changed. In contrast, the concentration of receptor C changed, while its affinity with ligand D was fixed. Fig 4b shows how AB interactions change along with receptor C concentrations under different affinities between receptor A and ligand B. The “X index” of the figure is the number of receptors C on cell surfaces. The relative increment of AB interactions, as receptor C changes from 0 to a given concentration, is recorded in the Y axis. The relative increment of AB interactions is calculated as $\left(N_{AB}^{C}-N_{AB}^{0}\right)/N_{AB}^{0}$, in which $N_{AB}^{C}$ is the number of AB interactions under a given concentration of receptor C, while $N_{AB}^{0}$ is the number of AB interactions without receptor C on cell surfaces. It offers a quantitative way to measure the relative increase of AB interactions towards cells that express higher levels of receptor C than normal. Therefore, the relative increment of AB interactions defines the specificity that ligand B recognizes the cells overexpressing receptor C. As a result, in addition to the positive correlation between receptor C concentration and increment of AB interactions, which has already been illustrated in Fig 4a and 4b further shows that the lower binding affinity between receptor A and ligand B enhances the relative increment of AB interactions, given the higher surface concentrations of receptor C. The figure thus indicates that, although relatively small numbers of interactions are formed between A and B when their binding affinity is low, these interaction are more sensitive to the change on concentration of receptor C. In another word, when a variety of ligands are exposed to cells with overexpressing surface receptors, our simulations suggest that the ligands with reduced affinity have higher specificity to distinguish these cells relative to the ligands with higher affinity. This is consistent with a previous study using Langevin dynamic simulation [34]. Of particular relevance is a recent experimental report using a chimera containing epidermal growth factor (EGF) as a cell targeting element and interferon-α-2a (IFNα-2a) as an activity element to initiate signal transduction [42]. This study demonstrated that mutations in the chimera that reduced the affinity between IFNα-2a and IFNα receptor 2 (IFNAR2) can bind to cells expressing EGFRs, while the same mutants of IFNα-2a monomers cannot. Moreover, the chimera afforded higher selectivity to cells expressing larger number of EGFRs relative to cells expressing fewer EGFRs. This EGFR-dependent effect is more evident when the affinity between IFNα-2a and IFNAR2 in the chimera was reduced. These experimental observations are quantitatively captured by our computational simulations. Consequently, the negative correlation between binding affinity and cell specificity suggested by our studies brings new insights to the rational design of macromolecular compounds as ligands to stimulate important cellular functions.

### Investigate the roles of binding site arrangement and conformational flexibility in binding of multivalent ligands

**Fig 5:**
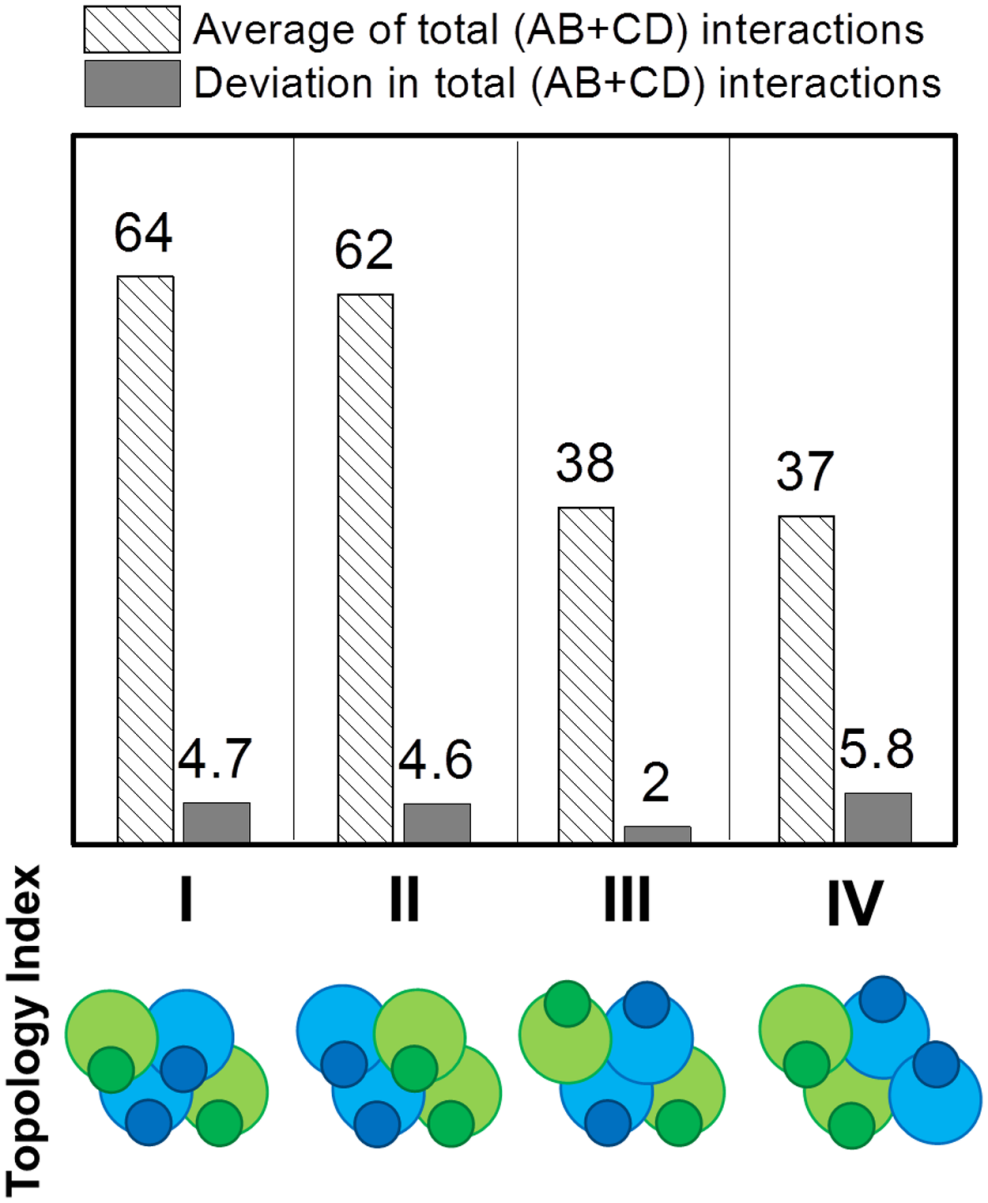
In order to investigate the functional role of binding site organization, four different topologies were designed. Each topology includes two ligands B and two ligands D, as shown in the bottom row. The binding of all four types of topology were simulated. The average numbers of interactions between ligands and receptors are plotted as striped bars, while the deviations in total number of interactions are plotted as black bars. The first two topologies show similar average and deviation. Moreover, the fourth model has higher deviations than the third model, although they have very close average number of interactions.

The overall binding properties of a tethered multi-specific ligand can be affected by variables/degrees of freedom other than stoichiometry and affinities. For instance, the precise spatial arrangement and overall architecture of tethered ligand assembly can have significant impact on the overall binding behavior. These topological constraints are naturally embodied in our rigid body modeling approach, and in principle, all possible combinations of spatial arrangement can be enumerated with a given number of binding sites and ligand types. To simplify the analysis, only representative models were considered. Specifically, four different topologies were examined for the multi-specific ligand assembly *B*_*2*_*D*_*2*_, as shown in the bottom row of Fig 5. In the first two models, binding sites of all four ligands are oriented in the same direction (downwards), but the relative packing arrangement between ligand B and D is different. In the remaining two models, two groups of binding sites are organized in an anti-parallel fashion (two upwards and two downwards). In the third model, the same types of ligands are in different orientations, while in the fourth model, the same types of ligands are in the same orientations. The binding of all four types of complexes were simulated. The average numbers of interactions between ligands and receptors are plotted as striped bars in Fig 5 for each topology, while the deviations from the average number of interactions are plotted as black bars. The first two models in the figure show similar averages and deviations. In contrast, the last two models show much lower number of interactions. When all binding sites are in the same direction, they can simultaneously engage multiple receptors. Notably, the fourth model has a higher deviation than the third model, although the average numbers of interactions are very similar, suggesting that the anisotropic arrangement of binding sites leads to higher fluctuations in binding. The asymmetry in ligand complexes cause they can only bind to one type of receptors at the same time. This releases the coupling effect between two types of receptors, which further results in the instability and higher fluctuations in binding. Considering that the total binding sites of a multi-specific ligand are the same for all four models, the differences of binding among different model reflected from our simulation results therefore indicate that, in addition to the number of binding sites, the spatial organization of ligands also plays an important role to regulate binding between receptors and ligands.

**Fig 6:**
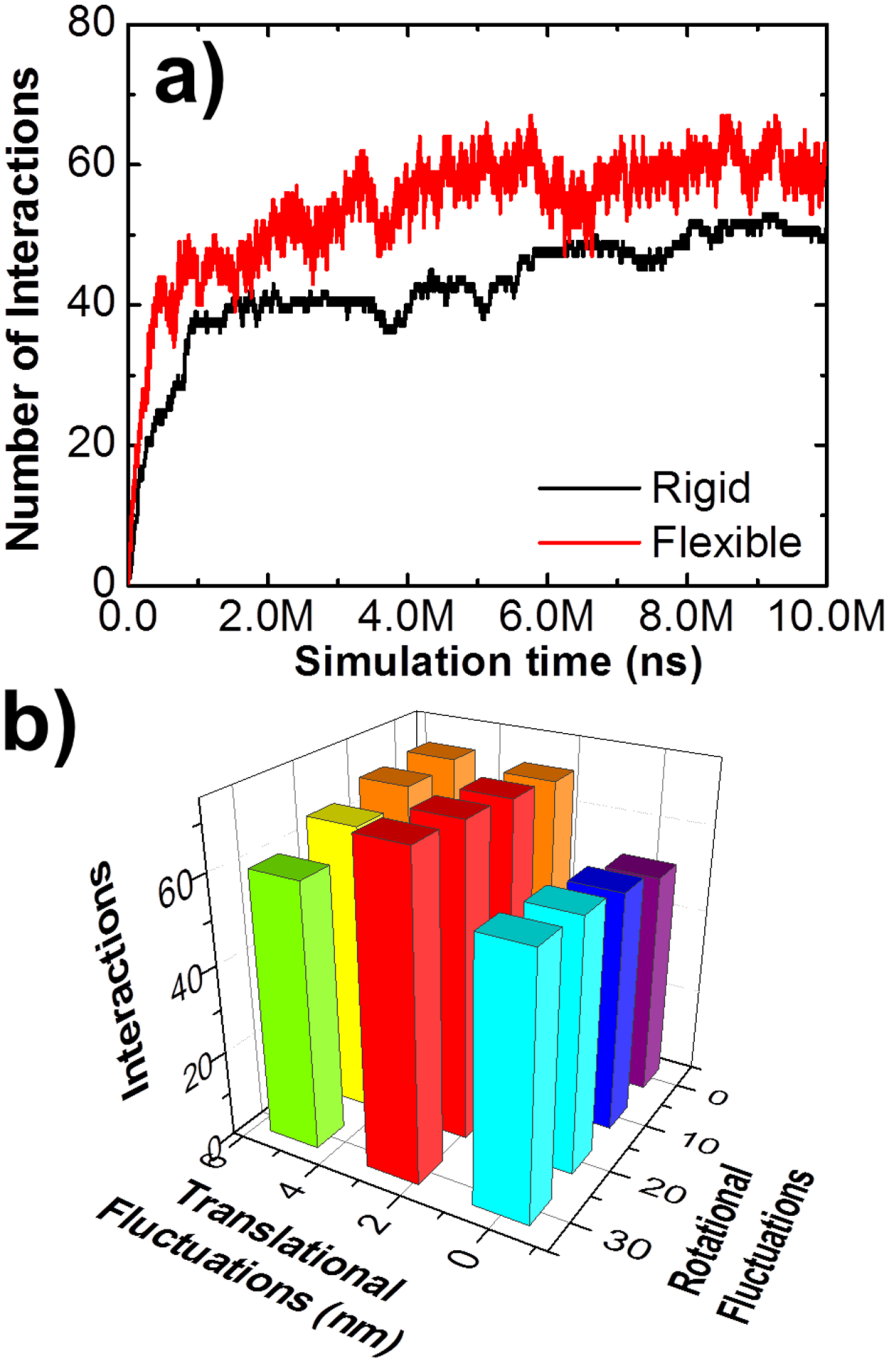
The internal flexibility of multi-specific ligands was incorporated in the simulations. Comparing a simulation in which flexibility was incorporated (red) with a simulation without flexibility (black), we found that flexibility not only leads to more interactions on average, but also causes larger fluctuations in the number of interactions along simulation time **(a)**. We further changed the maximal ranges of translational and rotational perturbations in each simulation step to adjust the spatial variability between different binding sites in a multi-specific ligand. The overall testing results are plotted in **(b)** as a three-dimensional histogram. The maximal ranges of translational and rotational fluctuations are indexed along the x and y directions. The figure suggests that the overall binding of ligands is promoted by the intramolecular flexibility within an appropriate range. However, binding will be negatively affected when molecules are over flexible.

Another important feature is the internal flexibility of a tethered ligand assembly, with flexibility defined as the small range of conformational fluctuations around a given topological arrangement. The flexibility of a tethered ligand assembly is incorporated in our simulation as spatial variations of each binding site relative to its equilibrium position. Specifically, within each simulation time step, an additional operation was added to generate a small random perturbation along three translational and three rotational degrees of freedom for each binding sites in a ligand assembly. Fig 6a gives the comparison between a simulation in which flexibility was incorporated (red) and a simulation without flexibility (black). The third scenario of ligand model *B*_*2*_*D*_*2*_ (Fig 1d) was used for both simulations and identical values were assigned for all other parameters. The figure shows that flexibility not only leads to more interactions on average, but also causes larger fluctuations in the number of interactions during the simulation. We also changed the maximal ranges of translational and rotational perturbations in each simulation step to adjust the flexibility of the entire ligand assembly. The maximal range within which each ligand binding site in a tethered assembly can be randomly rotated was set from 0 to 30 degrees with an interval of 10 degrees. The maximal range of translational perturbation was set from 0 to 6nm, with an interval of 2nm. Simulations were generated for all combinations and the interactions between ligands and receptors were calculated. The overall results are presented in Fig 6b as a three-dimensional histogram. The figure suggests that binding of a multi-specific ligand assembly is promoted by the appropriate selection of its intramolecular flexibility. If the molecule is overly flexible; however, binding can be negatively affected. Overall, these studies illustrate that topology and flexibility of a multi-specific ligand can be fine-tuned to optimize its binding with cell surface receptors.

## Discussion

Binding of multivalent molecules is a ubiquitous phenomenon in living cells. For instance, intracellular signaling platforms such as apoptosome contain multiple subunits to amplify downstream signal transduction [43]. The cascade of these signaling pathways is initiated by the activation of various cell surface receptors through binding with their extracellular ligands. Similarly, the engagement of cell surface receptors and ligands can be spatially and temporally regulated when extracellular ligands are organized into multivalent assemblies, called multi-specific ligands. To probe the functional role of this multi-specificity in ligand-receptor interactions, a rigid-body based computational model has been developed. The model attempts to realistically simulate the process of binding between receptors and ligands to the greatest extent. To achieve this goal, our previously reported diffusion-reaction algorithm has been enhanced. The new method confines the diffusion of membrane receptors to a two-dimensional surface, while ligands are free to diffuse above the cell surface in three dimensions. The multi-specificity of ligands was implemented by incorporating spatial tethering of different binding sites, which takes both homogeneous and heterogeneous oligomerization into account. Although the model is coarse grained, basic structural details for each receptor and each binding site in a ligand can be captured, such as rotational diffusion and geometric constrains during binding. Finally, the proper selection of model parameters such as molecular size, diffusion coefficient and binding affinities, maximize the biological utility of our simulation results.

One of our major observations is the coupling effect between avidity of multiple binding sites and affinity of individual binding sites. When the individual binding affinities are weak, ligands dissociate from receptors relatively soon after they associate. The life-time of a ligand-receptor interaction is much shorter than the average time of ligand diffusion before the ligand can encounter with its binding partner. In another word, a ligand is very likely to diffuse away from surface before it can rebind to the receptor that it originally binds to. In this case, the tethering of different binding sites causes little effect. Therefore, no coupling was observed between binding sites in a multi-specific ligand (the lower left corners in Fig 3). When the binding affinities increase to the intermediate range, on the other hand, the interaction between one binding site in a multi-specific ligand starts to affect the binding of other sites. More specifically, the life-time of this intermediate-strength interaction is comparable to the average time of diffusion a ligand takes before it can encounter with its binding partner. We speculate that this further causes the following effects. Firstly, binding between any binding sites in a multi-specific ligand with their receptors simultaneously brings other binding sites in the ligand close to cell surface. In another word, the local concentration of different binding sites is increased due to the spatial tethering. Therefore, if the ligand dissociates from its original receptor, it will bind to other receptors with higher probability. Similar phenomena have been observed in the multivalent lectin-glycoconjugate interactions [44]. Moreover, binding between any binding sites in a multi-specific ligand with their receptors causes the entire tethered assembly to diffuse together with the receptors on cell surface, which provide better orientation of other binding sites in the ligand to their receptors. Additionally, a multi-specific ligand will leave the plasma membrane only if all its binding sites dissociate from their receptors, which effectively decrease the overall dissociation rate. Consequently, we observed that the interaction between one binding sites in a multi-specific ligand strengthens the binding of other sites. This effect is more evident when the avidity in a multi-specific ligand is increased.

However, when the binding affinities further increase to the very strong range, interestingly, we found the negative coupling between different binding sites in a multi-specific ligand (the upper right corners in Fig 3). This may be the consequence of the following reason. The life-time of a strong ligand-receptor interaction is much longer than the average time of ligand diffusion before the ligand can encounter with its binding partner. Moreover, the two-dimensional diffusions of receptors on plasma membrane are much slower than the three-dimensional diffusions of proteins in solvent environments. As a result, the binding of different sites in a tethered ligand to their cell surface receptors becomes competitive. In another word, if one site of a ligand binds to its target receptor, it will take very long time for other unbound sites in the same ligand to find their target receptors, as the entire ligand-receptor complex diffuses on cell surfaces. Meanwhile, the long dissociation time of the ligand-receptor complex, due to the strong affinity prevents other sites from diffusing back into the three-dimensional extracellular space and binding to their corresponding receptors. It needs to be noted that this kinetic trapping effect does not change the overall thermodynamics of the system. Therefore, when simulations reach infinite time, we should observe that most ligand sites can ultimately bind to their receptors due to the strong affinities. However, the negative coupling due to the kinetic issue has more functional relevance in the context of understanding the role of spatial organization in multi-specific ligands, because these biological processes occur within the physiologically meaningful time scale. It is reasonable to assume that both increase of encounter probability and decrease of overall dissociation of a multivalent complex are proportional to its internal structural flexibility, which has been validated by the further simulations. Our computational studies therefore provide quantitative insight into the general principles governing the binding between multivalent ligands and surface-bound receptors. In the future, additional features will be integrated into the model for the application to specific biological systems. For instance, more specific information about structural fluctuations between different binding sites of a ligand and the binding constants of wild-type or mutated ligand-receptor interactions can be achieved by higher-resolution simulation methods such as Brownian dynamic simulation [45–54]. These data can be fed into the current rigid-body based model by the further development of a multi-scale framework. Finally, it is worth mentioning that in some cases, binding of one ligand-receptor pair might change the affinity of other ligand-receptor pairs due to the conformational changes of these molecules upon binding. This effect is called allosteric regulation [55]. However, since molecules were simplified by rigid-bodies, the conformational changes within each ligand and receptor cannot be reflected by our model. Therefore, the impacts of allosteric regulation on ligand-receptor interactions were not taken into account here. The principles revealed in this study are purely based on the spatial organization of multi-specific ligands.

Future applications of our model include the design of multi-specific ligands to recognize specific cell types based on the differentiated expression levels of their surface receptors. There exist large ranges of expression level for membrane receptors in different types of cells. For instance, expression of immune receptors on the surfaces of different T cells are highly variable, such that a wide spectrum of antigens can be targeted [56]. In cancer biology, specific mutations lead to the overexpression of certain receptors, such as cell adhesion molecules on membrane [57], which is a hallmark to distinguish tumor cells from normal cells [58]. Therefore, understanding the quantitative relation between ligand binding specificity and receptor expression level is important to maximize drug efficacy and minimize off-target drug toxicity. If a ligand is monomeric, its binding probability depends only on its concentration and the expression level of its target receptor. Interestingly, by linking the ligand into a dimeric complex in which the second ligand subunit binds to a receptor with stable expression on cell surface, we show that the binding specificity of the first ligand not only depends on the expression level of its target receptor, but is also modulated by the binding affinity of the second ligand. These results provide insights to the practical strategies of next-generation drug design. By generating multi-specific ligands with design principles based on binding affinity, topology of binding sites and expression levels of their cognate receptors, we will be able to control the selectivity of these ligands for specific cell types. Conjugating these ligands with traditional cancer drugs may enable delivery to the target tissue with a much higher selectivity and reduced off-target effects [59]. Similarly, the incorporation of T cell receptor-specific recognition modules into tethered ligand assemblies may allow for the selective induction or suppression of disease-relevant T cells [60]. The selectivity associated with such reagents may reduce the extensive side effects associated with nearly all biologics-based immunotherapies, which elicit global immune modulation of the entire T cell repertoire [61]. The practical development of such ligand complexes could pave the way for a new generation of engineered immunotherapies.

## Supporting information

S1 Fig The two-dimensional contour plots of total (AB+CD) interactions formed in the first, second and third simulation scenarios under all combinations of AB and CD affinities. (PDF)
