# Supplementary material for: General principles of binding between cell surface receptors and multi-specific ligands: A computational study
Source: PLoS Comput Biol. 2017 Oct 10;13(10):e1005805. doi: 10.1371/journal.pcbi.1005805 (PMC5654264; doi:10.1371/journal.pcbi.1005805)
Supplement: S1 Fig — (PDF) [file pcbi.1005805.s001.pdf]

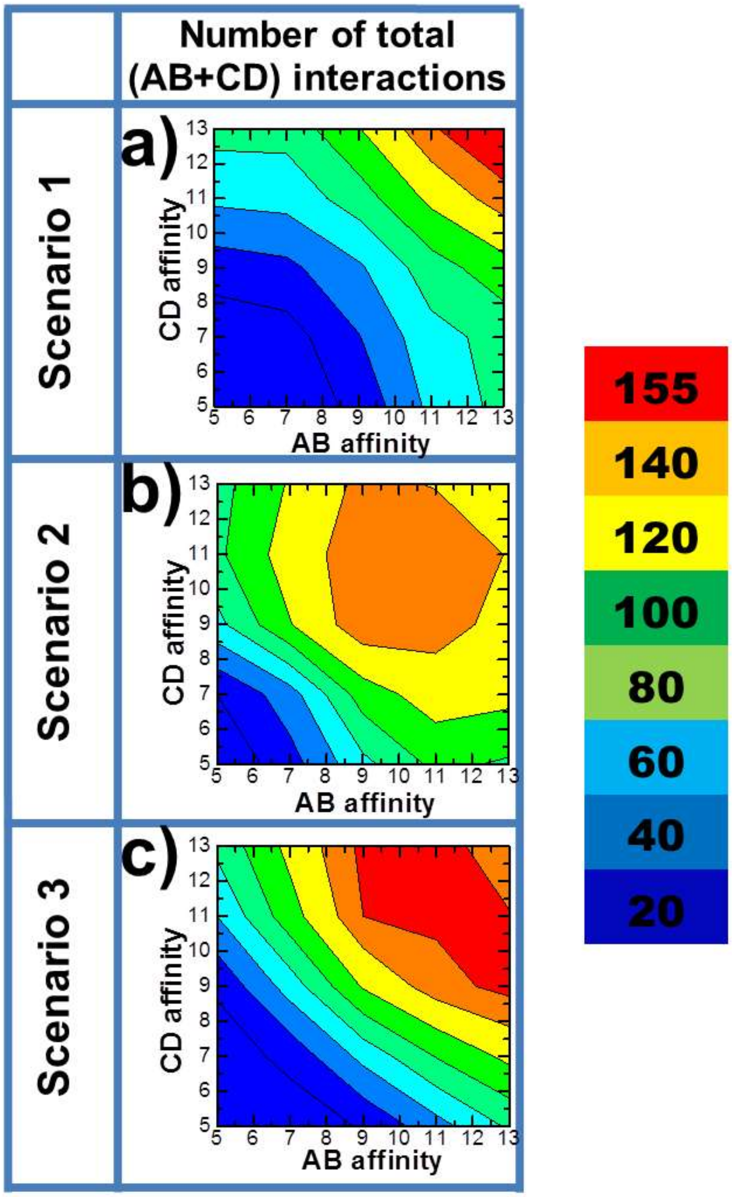

Figure S1: The numbers of total (AB+CD) interactions formed in the first, second and third scenarios are illustrated in **(a)**, **(b)** and **(c)** under all combinations of AB and CD affinities. The AB binding affinity is indexed along x axis, while the CD binding affinity is indexed along y axis. The color index of the contours indicates the number of interactions, as shown on the right side of the figure.
